# Improving the Patient Experience in Breast Reconstruction: ERAS and Beyond

**DOI:** 10.3390/jcm14155595

**Published:** 2025-08-07

**Authors:** Evan J. Haas, Bilal F. Hamzeh, Zain Aryanpour, Jason W. Yu, David W. Mathes, Christodoulos Kaoutzanis

**Affiliations:** 1 School of Medicine, University of Colorado Anschutz Medical Campus, Aurora, CO 80045, USA; evan.haas@cuanschutz.edu (E.J.H.); bilal.hamzeh@cuanschutz.edu (B.F.H.); 2 Division of Plastic and Reconstructive Surgery, Department of Surgery, University of Colorado Anschutz Medical Campus, Aurora, CO 80045, USA; zain.aryanpour@cuanschutz.edu (Z.A.); jason.yu@cuanschutz.edu (J.W.Y.); david.mathes@cuanschutz.edu (D.W.M.)

**Keywords:** breast reconstruction, mammaplasty, perioperative care, enhanced recovery after surgery (ERAS), patient-reported outcome measures (PROMs), patient-centered care, patient satisfaction, clinical protocols

## Abstract

**Background and Objectives**: Breast reconstruction after mastectomy has been shown to significantly improve psychosocial wellbeing and quality of life. Enhanced Recovery After Surgery (ERAS) protocols, especially those tailored to breast reconstruction, have revolutionized recovery by reducing complications, pain, opioid use, and hospital stay while improving patient satisfaction. The purpose of this narrative review was to present existing practices and supporting evidence within current ERAS protocols, as well as propose a modern ERAS framework centered around enhancing the patient experience following breast reconstruction. **Methods**: A focused literature search was conducted to identify studies investigating emerging approaches to patient care and surgical techniques adopted as part of a broader ERAS workflow **Results**: Some recent innovations include digital ERAS tracking, robot-assisted techniques, neurotization, and closed incision negative pressure therapy (ciNPT). These innovations show promise in reducing morbidity following reconstruction and may greatly improve sensory and functional outcomes. These advancements also reflect a shift toward more holistic, patient-centered care, extending beyond immediate clinical needs to address long-term wellbeing through psychosocial support and patient-reported outcome measures. Incorporating tools that validate patient perspectives helps guide interventions to optimize satisfaction and recovery. **Conclusions**: Future research should aim to standardize ERAS protocols by incorporating evidence-based practices, reinforcing breast reconstruction as a patient-centered, evidence-driven process that is focused on comprehensive recovery and improved quality of life.

## 1. Introduction

Female breast cancer has emerged as the most commonly diagnosed malignancy, impacting 2.3 million women and leading to over 600,000 deaths globally each year [1]. In the United States, approximately 310,720 new cases of invasive breast cancer were diagnosed, and 40,000 women were estimated to die from breast cancer in 2024 alone [2]. The gold standard treatment for early breast cancer diagnoses includes breast-conserving surgery (BCS) that is typically combined with radiation therapy (RT). Mastectomy is another common option, which is either a personal choice or necessary for patients with contraindications to BCS, such as cases with multicentric disease, prior radiation, and genetic predispositions. Notably, the recent literature has demonstrated that patients who undergo breast reconstruction following therapeutic treatment report better psychosocial and aesthetic outcomes compared to those who have BCS or mastectomy alone [3].

Around 60 years ago, Cronin and Gerow introduced the silicone breast implant, marking a pivotal moment in the history of breast reconstruction [4]. For more than a decade, few significant advancements followed until 1977 when Schneider, Hill, and Brown introduced the latissimus dorsi musculocutaneous flap, marking the beginning of autologous tissue breast reconstruction [5]. Just a few years later, Radovan broadened the range of eligible patients with the development of tissue expanders [6]. As a result, patients with more extensive skin deficits could pursue breast reconstruction, an option that was previously unavailable to them. Implant-based and autologous tissue breast reconstruction, therefore, represent the two principal approaches to post-mastectomy breast reconstruction today. Implant-based reconstruction offers a less invasive approach than autologous tissue breast reconstruction and remains the most utilized form of postmastectomy breast reconstruction in the United States, with some sources finding it accounts for nearly 70% of all cases [7]. Procedures vary widely, including by plane of implant placement (prepectoral or subpectoral) and use of adjunctive techniques such as acellular dermal matrices and fat grafting, which have cumulatively fostered improved aesthetic outcomes and patient satisfaction [8,9]. Conversely, autologous tissue breast reconstruction involves a more complex procedure utilizing the patient’s own tissue to reconstruct the breast. Procedures vary by donor site, with the deep inferior epigastric perforator (DIEP) flap, an abdominal tissue flap, being the most commonly used and representing the current gold standard [10]. Although autologous tissue breast reconstruction is associated with greater postoperative pain, longer hospital stays, and increased risk for tissue and microvascular morbidity, patients benefit from distinct advantages, including improved tactile quality, superior aesthetic durability, and reduced long-term revision rates [11,12,13,14].

The landscape of breast reconstruction has evolved significantly in recent years. In addition to a substantial increase in case volume and a shift towards immediate and autologous reconstruction, there has also been a paradigm shift from an exclusive focus on technical and operative outcomes (i.e., complications) toward a more holistic approach that emphasizes perioperative education, patient-reported outcome measures (PROMs), and recovery optimization [15]. Enhanced Recovery After Surgery (ERAS) protocols have emerged as a major area of ongoing research, aiming to improve perioperative pain control through multimodal analgesia, facilitate early ambulation, and expedite return to baseline function following extensive reconstructive procedures [16]. These protocols provide an evidence-based approach to perioperative care that begins well before the patient enters the operating room. This narrative review will explore current approaches to breast reconstruction, focusing on both implant-based and autologous tissue techniques, while examining how ERAS protocols and recent innovations continue to transform the field of breast reconstruction.

### Overview of Enhanced Recovery After Surgery (ERAS)

In 1997, Danish physician Dr. Henrik Kehlet first described a multimodal approach to enhanced postoperative recovery [17]. This led to the introduction of what are now called ERAS protocols, which were coordinated measures before, during, and after surgery to reduce common complications of surgery and identify risk factors leading to these increased complications.

Today, ERAS is a consensus-driven and evidence-based perioperative protocol intended to reduce length of hospitalization, limit pain and opioid use, improve outcomes, and enhance patient satisfaction. ERAS was initially driven by the idea that recovery and outcomes could be improved, and that a wide breadth of data could be leveraged in the development of standardized and broadly adoptable protocols to this end.

In 2017, a consensus review by Temple-Oberle et al. was published, representing the official ERAS society recommendations pertaining to the perioperative management of patients undergoing breast reconstruction specifically [18]. This consensus review describes 18 core ERAS items intended to limit hospital length of stay (LOS), postoperative pain and opioid use, and complications—serving to maximally enhance patient experience. These 18 items span the entire perioperative period and require a multimodal, multidisciplinary team-based approach to the management of patient care before the operation and after their hospital discharge, both within and beyond the operating room or clinic.

Following the publication of their guidelines in 2017, multiple other studies have since demonstrated the utility of ERAS protocols in the context of breast reconstruction, finding reduced LOS and opioid use in both implant-based breast reconstruction (IBR) and autologous breast reconstruction (ABR) [16,19].

The key pillars of ERAS, adapted from Temple-Oberle et al. and outlined in Table 1, include multimodal pain management to reduce opioid consumption, anesthesia tailored toward the reduction in pain and nausea, minimal preoperative fasting and early feeding, and early postoperative ambulation.

## 2. Methods

Co-authors discussed key aspects of the current use of ERAS protocols in breast reconstruction, outlined in Table 1, which warranted further investigation. These items are outlined in the Section 3 and Section 4. Upon identification of these items, a targeted literature search was conducted on several databases, including PubMed, Embase, and Web of Science, to identify relevant articles investigating the use of and supporting evidence behind established and emerging approaches to patient care and surgical techniques adopted as part of a broader ERAS workflow. Findings were synthesized into a newly proposed ERAS framework based on supporting evidence and potential for improving the patient experience in breast reconstruction.

## 3. Results

### 3.1. Evidence Supporting ERAS in Breast Reconstruction

Several studies have demonstrated the utility of ERAS in pain management and reducing LOS in IBR, implant-based breast augmentation, and ABR patients. A summary of these findings is outlined in Table 2. In IBR patients specifically, ERAS protocols have demonstrated superiority in reducing LOS and postoperative pain and opioid requirements, facilitating broad improvements in the perioperative patient experience [20,21]. One study published in 2020 showed that ERAS protocols utilizing non-opioid analgesics, including gabapentin and nonsteroidal anti-inflammatory drugs, significantly shortened LOS and reduced opioid intake during the two-day postoperative period by approximately five-fold overall in patients undergoing mastectomy with immediate subpectoral IBR [22]. Niu et al. compared patients following traditional inpatient protocols with those following ERAS-driven same-day discharge protocols in staged IBR with tissue expanders and found no differences in major complications or reoperation rates, highlighting the safety of same-day discharge ERAS protocols in IBR [20]. Beyond reducing LOS, one study has also shown that ERAS protocols can significantly reduce the rate of unplanned admissions in patients undergoing IBR relative to traditional protocols, further supporting the safety and efficiency of outpatient IBR [23]. We recently conducted a randomized controlled trial that showed that same-day discharge, compared to overnight admission following ERAS pathways in patients undergoing mastectomy with immediate IBR, resulted in similar rates of complications, pain, and analgesic use, with minimal differences in patient-reported outcome measures (PROMs) and an estimated cost reduction of over threefold [24].

ERAS protocols also demonstrate superiority in aesthetic breast augmentation surgery. Stahl et al. assessed the implementation of systematic ERAS protocols incorporating preoperative patient education, perioperative analgesia and anesthesia, including intercostal blocks, and immediate postoperative ambulation, rapid discharge, and regular communication in patients undergoing aesthetic breast surgery [25]. They found significant improvement across all BREAST-Q PROM domains in breast augmentation surgery patients, further showing patient expectations were met with respect to how soon they would be able to engage in physical activities postoperatively. Enhanced Recovery After Breast Augmentation Surgery protocols standardizing parameters, including non-opioid pain management, anesthesia, nausea prophylaxis, and early ambulation, have been associated with early discharge within 2 h after surgery and rapid return to functional baseline within 24 h in 95% of patients [26]. Other work corroborates the efficacy of ERAS in facilitating safe outpatient aesthetic breast surgery, reducing prescribed opioids by 41.5% [27].

ERAS pathways have seen broader adoption in ABR relative to IBR, with several studies showing it affords improvement with LOS, postoperative pain, and opioid use, and in patient experiences relative to traditional protocols [28]. This is likely due to the fact that ABR procedures are generally longer and more invasive than IBR procedures, often necessitating inpatient hospitalization for postoperative pain management, close monitoring, and functional rehabilitation prior to discharge, highlighting the need for enhanced recovery. Shortly after publication of the 18-item ERAS consensus review in 2017, the Temple-Oberle et al. group published ERAS guidelines for ABR, including pathways for analgesia, antimicrobial and antiemetic prophylaxis, diet, fluids, intraoperative management, and activity [18,29]. Relative to the traditional pathway group, ERAS pathway patients showed significantly reduced intravenous opioids by almost 9-fold and postoperative narcotics by almost 3-fold. ERAS patients were also shown to have significantly lower pain scores postoperatively. Moreover, ERAS patients showed reduced antiemetic use by almost 3-fold and had resumption of regular diet, ambulation, and discharge significantly sooner than patients in the traditional pathway cohort. Notably, all patients were shown to have similar complication rates, marking the safety and efficacy of ERAS in ABR. Other studies have also corroborated these findings, suggesting the need for wider adoption of ERAS in ABR as the standard of care, making the recovery experience in ABR less burdensome and more attractive to patients [16]. Muetterties et al. showed that 96.0% of ERAS patients were discharged by postoperative day 3, while 88.9% of traditional recovery patients were discharged by postoperative day 4 [30]. They further demonstrated that both inpatient opioid administration and prescribed milligram morphine equivalents (MMEs) at discharge saw over a two-fold reduction in the ERAS group compared with traditional recovery patients. Haddock et al. corroborated these results in a cohort of patients undergoing abdominally based ABR with deep inferior epigastric perforator (DIEP) flaps, also finding total hospital MME to be reduced by over a factor of two in the ERAS group compared with control patients [31].

Many studies also note significant cost savings and improved margins per patient through utilization of ERAS protocols in microsurgical ABR, supporting the idea that patient-centric improvements often coincide with economic value. One study conducted by Oh et al. found that, compared with traditional protocols, implementation of ERAS protocols resulted in adjusted mean cost reductions of USD 4576 [32]. Another study showed that ERAS protocols had a significantly improved cost-effectiveness ratio, with ERAS-driven postoperative wound management and drain care reducing cost by USD 3200 compared with traditional protocols, and ERAS analgesia protocols further reducing cost and LOS [33]. Our own study showed that through minimizing LOS and through a four-fold reduction of postoperative opioid use, ERAS pathways were associated with downstream cost savings of USD 4400 per patient undergoing ABR [34].

Looking beyond the current state of ERAS and into the potential for future refinement, there are several changes that have been implemented across various surgical disciplines toward the improvement of workflows and the optimization of patient-centered algorithms. These changes include the implementation of digital applications that can be used to track the ERAS algorithm in improving autonomy, uniformity, compliance, and self-management for patients, as well as improving tracking of patients and postoperative outcomes for healthcare providers. Moreover, ERAS can be further adapted to provide patient-specific care in a still operationalized manner, tailoring perioperative management to individual patient risk factors. Some examples may include emphasizing glucose control in diabetic patients or improving preoperative psychosocial support in patients experiencing elevated anxiety, improving individualized care, and maximizing patient agency and education.

### 3.2. Evidence Supporting the Use of Patient-Reported Outcomes Measures

With individual, subjective patient experience at the core of breast reconstruction, the development and utilization of standardized subjective measures is crucial. Traditional surgical metrics such as complication rates and surgeon-reported aesthetics do not capture patient-centered domains such as improvements in body image, psychological well-being, or satisfaction. PROMs provide an operationalized method through which to evaluate these parameters. Among them, the BREAST-Q is the most widely used and validated instrument. Originally described by Pusic and colleagues in 2009 after recognition of the paucity of psychometric evidence-based PROMs validated for use in breast reconstruction, the BREAST-Q outlines two domains, satisfaction and quality of life, each with three subdomains that further characterize patient experience in the context of breast reconstruction [35].

BREAST-Q is frequently used to assess patient experiences across breast reconstruction procedures and over time postoperatively. One prospective cohort study showed that ABR yielded higher “Satisfaction with Breasts” scores compared to IBR, with “Physical Well-Being” scores continuing to improve in both groups when reassessed five years postoperatively [36]. Moreover, other studies have mirrored these results, showing that DIEP flap reconstruction had higher patient satisfaction scores than direct-to-implant IBR patients, and demonstrated that only DIEP patients continued to see improvements in satisfaction and sexual and psychosocial metrics > 1 year postoperatively [37]. The BREAST-Q has also demonstrated utility in assessing the role of subtype of reconstruction, follow-up periods, and staging on patient satisfaction [38]. Outside of BREAST-Q, there has also been investigation of other PROMs pertaining to subjective measures such as pain, discomfort, and sensation, including the Breast Sensation Assessment Scale (BSAS), the Breast Cancer Treatment Outcomes Scale (BCTOS), BRECON-31, EORTC QLQ-BRECON-23, EQ-5D-5L, ICECAP-A, and Vanderbilt Mini-PROM-Breast, among others [39,40,41,42]. Our study assessed patient attitudes toward their recovery utilizing the QoR-15 and assessed measures pertaining to domains of pain interference, depression/sadness, participation in activities, fatigue, and anxiety/fear using the PROMIS-29, which allowed for the appreciation of subtle differences in patient experiences and perceptions based on discharge day [24,43,44]. No significant differences were observed in QoR-15 outcomes, indicating similar perceived quality of recovery and support, while PROMIS-29 showed that same-day discharge resulted in worse physical function scores (39 vs. 42, *p* < 0.05) and more sleep disturbance (55 vs. 50, *p* < 0.05) relative to the patients admitted on postoperative day one. Though the clinical significance of these differences is unclear, these findings do demonstrate the clear utility of PROMs in operationalizing and contextualizing granular aspects of the patient experience to foster continuous improvement of breast reconstruction. BREAST-Q and other PROM questionnaires have also been used to compare outcomes in ERAS pathways, but their implementation in the literature and clinical setting is not yet standard procedure.

The utility of PROMs extends beyond comparison of surgical methodologies and clinical approaches in research; they are increasingly being used to counsel patients and help practitioners navigate shared decision-making to enable patient-centered care. Regulatory and reimbursement trends in the US are also placing greater emphasis on PROMs as quality metrics [45,46].

## 4. Discussion

### Future Directions: ERAS and Beyond

While the implementation of ERAS protocols has led to significant benefits for hospitals, surgeons, and most importantly, patients, one would argue that there is still an opportunity to improve the overall experience for patients undergoing breast reconstruction. Currently, the framework of most protocols is temporal, categorizing interventions into preoperative, intraoperative, and postoperative. Rather than segmenting care in that manner, a more comprehensive model that emphasizes four experience-driven pillars can be established: Prepare, Protect, Restore, and Empower, outlined in Table 3.

This framework retains the foundational elements of the current, validated ERAS protocols, including preoperative counseling, sterility, fluid management, as well as postoperative multimodal pain control and early ambulation, and introduces emerging interventions that may offer a more holistic approach to reconstruction.

**Prepare**

Before surgery, patients are often told they must “prepare” for breast reconstruction. Patients are encouraged to improve their cardiovascular health, control their weight, and stop drinking alcohol or using tobacco and marijuana. These recommendations are not futile. However, no two patients are the same. Having a uniform preoperative approach to breast reconstruction fails to account for the status of an individual patient’s current health, risk factors, and needs. By identifying these factors, patients can gain greater insight into their own health, and care teams can offer personalized, evidence-based recommendations.

One promising avenue to achieve this level of precision is through the incorporation of digital modalities into ERAS pathways. With the central role that technology plays in the current state of medicine, it is imperative that the future of ERAS and breast reconstruction, in aligning with a patient-centered experience, leverages digital applications to support individualized care [47]. Interestingly, one prior study found that as many as 90,000 health-related applications were added to app stores in 2020 alone. Several studies have supported the utility of such applications in surgical patients specifically, finding their use to provide personalized medical care while being associated with feelings of increased safety and ameliorating issues resulting from inconsistent information, excessive trips to the clinic, and difficulty accessing information [48,49,50,51].

Incorporation of digital ERAS systems has also been shown to enhance patient safety through improving both patient and provider education and adherence to indicated protocols spanning the entire perioperative period [52]. Gopwani et al. described the implementation of a digital ERAS notification system in breast reconstruction that improved preoperative ERAS protocol adherence from 16% to 44% (*p* < 0.001), significantly improving metrics such as adhering to gabapentin and celecoxib administration (*p *< 0.001 and *p* = 0.006, respectively) [53]. Overall, the implementation of digital applications in ERAS for breast reconstruction has significant potential to improve patient experience by enhancing feelings of autonomy and education while maximizing adherence, in addition to limiting cost burden. However, limitations to digital ERAS modalities include potentially steep patient learning curves and difficulties with adopting use of technologies, challenges with provider adherence, and establishment of initial confidentiality-compliant digital infrastructure. Moreover, the use of additional, adjunctive digital platforms containing protected health information increases the risk of confidentiality breaches for patients.

In parallel, researchers have begun to implement artificial intelligence and machine learning to appropriately stratify patients by risk factors and predict complications, which offers further opportunity to optimize preoperative patient management. In a study by Chen et al., researchers developed a machine learning algorithm capable of evaluating preoperative risk and predicting postoperative complications of patients undergoing IBR [54]. While further validation must be done, these results do indicate that the incorporation of mobile applications and intelligence models may allow us to accurately identify patients who are at risk for certain complications and adjust their operative protocols proactively.

**Protect**

After patients are appropriately analyzed and have their protocols adjusted to fit their risk profile, the next priority becomes minimizing morbidity and optimizing outcomes inside the operating room. Most ERAS protocols tend to focus on fluid management and temperature control, which are crucial to reduce the risk of surgical site infections, wound healing complications, and ischemia time [55,56]. One study reported that patients who experience intraoperative hypothermia experienced rates of surgical site infections more than double (34.4% vs. 17%) that of patients who were normothermic intraoperatively. While these should not be foregone, the evolution of surgical technology and techniques now offers further opportunities to enhance intraoperative approaches and reduce the patient’s postoperative burden.

Breast reconstruction relies greatly on a surgeon’s ability to preserve soft tissue, vascularity, sensory function, and aesthetic appearance. For this reason, minimizing morbidity to donor and recipient sites in ABR is essential, not only to prevent complications but also to preserve and restore cosmesis.

In DIEP free flap harvesting, significant exposure and manipulation of the abdominal wall can increase the risk of hernias, bulges, chronic pain, and limited function postoperatively [57,58]. As such, data demonstrate the rate of abdominal complications to be as high as 16.7% following DIEP flap procedures, which could be underreported, and certainly contribute to significant postoperative morbidity [59,60,61]. A prior study implemented the BREAST-Q following DIEP flap breast reconstruction and found that 7% of patients reported abdominal weakness, further finding a statistically significant difference in the incidence of bulging and hernias in patients who underwent nerve sparing versus nerve sacrificing procedures [57]. Adjunctive approaches, such as the use of mesh, have shown promise in reducing the rate of such complications. Our own recent study using ovine-derived hybrid bio-synthetic mesh in flap donor site closure of DIEP patients, for example, resulted in a lower incidence of abdominal bulging compared with the non-mesh cohort (0% vs. 5.3%, *p *= 0.04) without increasing complication rates [62]. However, more broadly, attempts to reduce donor site morbidity through reduction in pedicle length and fascial incision have historically not been widely adopted due to the increased margin of error, especially for surgeons with limited experience [63].

Alternative minimally invasive approaches to dissection of the pedicle, such as laparoscopic and robotic approaches, have emerged and demonstrate mitigation of some of these issues without sacrificing pedicle length. In 2018, Hivelin et al. reported the first clinical case of a laparoscopic DIEP flap, demonstrating that it is possible to successfully perform ABR while greatly reducing trauma to the donor site [64]. Moreover, Shakir et al. described total extraperitoneal laparoscopic harvesting of single-vessel DIEP flaps with favorable outcomes [65]. Mean fascial incision lengths were 2 cm compared with traditional free dissections with incision lengths of up to 13 cm. The authors also report a mean length of hospital stay of 2.4 days compared with the standard 4-day course for free dissection and found that nearly 60% of patients avoided postoperative opioids entirely. Several other studies have further demonstrated broad applications of laparoscopic or robot-assisted DIEP flap breast reconstruction, also showing potential for the reduction of postoperative pain and donor site morbidity in breast reconstruction [66,67,68]. Notably, Lee et al. found that although there was a significant increase in the mean reconstruction time for the single-port robotic DIEP group compared with the traditional DIEP group (*p* < 0.001), there was significantly reduce intensity of postoperative pain (*p* = 0.001), intravenous opioid use (*p* = 0.003), and length of hospital stay (*p* = 0.002) with otherwise comparable rates of outcomes [69]. However, limitations to the adoption of minimally invasive approaches to breast reconstruction include high initial institutional cost, steep provider learning curve, and potential for both patient and provider hesitancy with the adoption of new surgical technologies.

Pain management is another potential area of focus in further refining and standardizing ERAS protocols. The adoption of certain approaches to analgesia in breast reconstruction is provider and institution dependent, with no standardized algorithm and few identified studies to date directly comparing the efficacy of various pain management protocols in the context of ERAS pathways. Peripheral nerve blocks have been increasingly utilized across various breast surgery procedures, including breast reconstruction, to mitigate postoperative pain and enhance recovery [70,71]. Considerable effort has been dedicated to identifying the most effective type of peripheral nerve block for reducing postoperative pain in breast reconstruction, with varying results from multiple studies. Peripheral nerve blocks used in breast surgery and reconstruction predominantly include local blocks at the abdominal donor site (i.e., rectus sheath, transverse abdominis plane or TAP) and recipient sites (pectoral blocks), as well as regional paraspinal and erector spinae blocks.

There is significant variation in the literature on the efficacy of abdominal peripheral nerve blocks on postoperative pain-related outcomes in this population [72]. While some studies suggest that abdominal peripheral nerve blocks reduce postoperative opioid usage and hospital LOS, most other studies found no difference in patients who did and did not receive blocks [73,74,75]. Our own data analyzing 225 patients has demonstrated that transverse abdominis plane and rectus sheath blocks do not reduce LOS and postoperative pain in DIEP free flap patients, suggesting they may not be as effective as other types of peripheral nerve blocks, such as pectoral blocks [76].

Studies analyzing pectoral nerve blocks in breast surgery patients have found reductions in postoperative opioid consumption, pain, and length of hospital LOS in breast reconstruction patients. Conti et al. found pectoral nerve blocks synergized with ERAS pathways in mastectomy patients to significantly reduce postoperative opioid use (*p* < 0.002), pain scores (*p *< 0.005), and LOS (*p *< 0.003) compared with patients solely undergoing general anesthesia [77]. Cylwik et al. had similar findings, showing that pectoral nerve blocks significantly reduced pain (*p *< 0.001), postoperative opioid use (*p* < 0.001), and LOS in the postoperative care unit in patients who had undergone mastectomy (*p *< 0.001) [78]. Notably, patients receiving pectoral nerve blocks in this study also experienced improved satisfaction relative to controls. However, the timing and administration of pectoral nerve blocks vary widely, with some performed preoperatively by anesthesia or pain specialists and others administered intraoperatively by the surgical team. This variability is further complicated by the significant denervation that occurs following mastectomy, which can alter postoperative pain perception irrespective of whether reconstruction is immediate or delayed.

Recent efforts have shifted to evaluate the impact of regional peripheral nerve blocks such as erector spinae and paravertebral blocks in breast reconstruction [79,80]. Both regional block types have shown promise in reducing postoperative pain and hospital LOS. Additionally, according to the gate theory of pain, analgesia should be administered prior to surgical incisions to optimally reduce pain; therefore, ERAS protocols should focus on peripheral nerve blockade in the immediate preoperative period [81]. While local nerve blocks at the abdomen and chest target somatic peripheral nerves, regional paraspinal and erector spinae blocks target both somatic and sympathetic nerves that provide sensation to the chest wall and abdomen, further enhancing their use in breast reconstruction and especially in abdominally based ABR procedures [82].

These data suggest that the adoption of more standardized protocols for analgesia as part of a uniform ERAS algorithm can serve to further maximize patient outcomes and protect patients from the adverse effects of excessive postoperative pain and opioid consumption.

**Restore**

Breast reconstruction, regardless of the type or timing, is a significant operation. In most cases, a substantial amount of breast tissue is removed to minimize the risk of recurrent malignancy, increasing the risk of significant aesthetic and functional deficits. The goal of reconstruction, however, is to restore form, function, and, more recently, sensation. Traditional advancements in reconstruction have been geared towards improving aesthetic outcomes. Now, emerging techniques emphasize restoring the sensory integrity of the breast and optimizing wound healing in breast reconstruction.

Two key innovations in this space include neurotization of abdominal-based free flaps in ABR and neurotization of the nipple areolar complex in nipple-sparing mastectomies in IBR. With regards to neurotization of flaps, in the past decade, mounting evidence has begun to support the efficacy of nerve coaptation in both objective and PROMs. The results of a recent double blind prospective trial demonstrated that touch thresholds were lower in innervated breast flaps than non-innervated flaps (47.8 vs. 71.2 g/mm^2^, *p* = 0.036), and that heat was less perceptible in non-innervated flaps than in innervated flaps (42.1% vs. 10.3%, *p* = 0.004) [83]. Of 29 innervated flaps included in this study, no adverse events were recorded. A second study, which included 94 innervated DIEP flaps, found similar results, but notably, identified that prior radiation to the breast did not significantly correlate with worse sensory recovery [84]. This finding is particularly noteworthy, as prior radiation therapy has historically been associated with poorer outcomes and impaired healing in breast reconstruction [85]. Although it does not negate the recognized adverse effects of preoperative radiation, this result suggests that nerve coaptation may help mitigate radiation-induced sensory deficits. Most importantly, these anticipated benefits of coaptation translate to improved PROMs. In a non-randomized prospective cohort study, patients with innervated DIEP flaps were significantly more likely to report good to very good sensation (26.2% vs. 10.9%, *p* = 0.041), a reconstructed breast that felt almost completely normal (16.9% vs. 3.6%, *p* = 0.019), and ongoing sensory improvement over time (67.7% vs. 41.8%, *p* = 0.002) [86]. These findings suggest that nerve coaptation in ABR not only improves objective measurements of maintained or restored sensation but also improves the perceived sensation and naturalness of innervated breasts.

With regards to neurotization of the nipple-areolar complex, the results are similarly supportive. Guido et al. demonstrated that most patients who underwent nipple-sparing mastectomies with neurotization of the nipple-areolar complex initially experienced a marked decline in sensation in the early postoperative period, with a gradual return to near-baseline levels by one year [87]. Another retrospective study by Tevlin et al. found that reinnervation during nipple-sparing mastectomy with autologous reconstruction significantly improved breast (4.8 g vs. 5.4 g) and areolar (4.84 g vs. 5.68 g) sensation and preserved nipple sensation compared to significant sensory loss in controls (*p* = 0.0001) [88]. Additionally, the amount of time to complete neurotization intraoperatively ranges from 8 min to 38 min, just a fraction of the operation time usually allotted for ABR procedures [89,90,91]. In contrast, implant-based reconstructions are shorter operations, and the additional time required to perform neurotization represents a proportionally larger increase in operative time, which should be acknowledged when comparing techniques. Collectively, these findings indicate that incorporating neurotization into ERAS protocols may yield meaningful clinical benefits while adding only minimal operating time.

Another intervention that has only recently made its way into breast reconstruction is closed incision negative pressure therapy (ciNPT). The most common negative pressure systems are called wound “VACs”, short for vacuum-assisted closure. They work by applying negative pressure over closed incisions, like those left behind at DIEP donor sites, to uniformly distribute edge tension towards the center of the wound, thereby protecting against wound dehiscence, bacterial invasion, and fluid collection such as seroma or hematoma [92,93,94]. It is thought that the application of subatmospheric pressure increases capillary diameter, stimulates angiogenesis, and expedites the growth of granulation tissue, thereby enhancing perfusion of difficult-to-heal wounds [95,96,97].

Prior to their use in breast surgery, wound VACs have been used in many other surgical settings to aid healing in complex open fractures, diabetic ulcers, and burns [98,99]. In 2006, Stoeckel et al. were among the first to apply VAC technology to complex breast wounds and reported a clinical benefit [100]. However, their retrospective study lacked statistical comparison, limiting the significance of their findings. Over a decade later, in 2018, Gabriel et al. reported that when negative pressure therapy was applied over breast incisions following postmastectomy breast reconstructions, patients experienced lower rates of surgical site infection, wound dehiscence, necrosis, and seromas when compared to the group that received standard of care dressings postoperatively [101]. Just two years later, a large systematic review by Cagney et al., which included 904 patients and 1500 total breast incisions, supported the results of Gabriel et al., finding that ciNPT significantly lowered the rate of total wound complications, surgical site infections, seroma, and wound dehiscence and necrosis [102]. And to further support their use in the healing of the reconstructed breast, Pieszko et al. demonstrated similar effectiveness in a randomized control trial, also adding that they improved skin elasticity over time at the incision [103]. There is high-level supporting evidence behind the efficacy of ciNPT in optimizing breast outcomes in breast reconstruction. However, the same level of support is not seen for the use of wound VACs on abdominal incisions following ABR. Moreover, the use of ciNPT is not without potential for adverse consequences. Patients can face complications such as loss of seal integrity requiring VAC resealing, superficial wound dehiscence, and dermatological reactions, including blistering erythema and pruritus refractory to oral antihistamines that can require VAC discontinuation [104].

A few studies, particularly the randomized control trials reported by Muller-Sloof et al., demonstrate a statistically significant reduction in abdominal wound dehiscence rates in patients who received a wound VAC postoperatively [105,106]. Additionally, a retrospective review by Siegwart et al. demonstrated a reduction in major surgical site complications in patients who received ciNPT compared to those who did not, but no individual complication [107]. While promising, many investigations of the use of abdominal incisions have observed sporadic or isolated benefits that are not reproduced across other studies. As an example, two recently published retrospective studies, one by Haas et al. and the other by Wang et al., both reported a trend of reduced complications across nearly all outcomes for patients who received wound VACs but did not identify any statistical significance [108,109]. While the use of ciNPT and wound VACs has shown promise in the healing of breast incisions, their exact effect on the healing of autologous donor sites is yet to be truly quantified. However, given the current literature, which is generally very supportive with few reported drawbacks, ciNPT shows considerable promise for improving wound healing, reducing surgical site complications, and possibly contributing to an improved patient experience postoperatively.

Advances in neurotization and ciNPT exemplify a recent shift in reconstructive philosophy. At one point, “macroscopic” safeguards such as intraoperative angiography transformed flap safety by allowing a real-time assessment of perfusion, dramatically reducing flap loss. As modern surgical outcomes have become reliable, the focus of reconstruction has shifted. It is no longer solely about repairing a defect left by oncologic excision, but rather focused on restoration of the breast to its original sensibility and function. “Microscopic” refinements such as neurotization and ciNPT embody this evolution.

**Empower**

Given the established value of clinically validated PROMs like the BREAST-Q in capturing the subjective patient experience, it is essential that PROMs be integrated as a standard component in the evaluation of breast reconstruction and ERAS pathways. Biswas et al. found that only 24.6% of the original literature published pertaining to breast reconstruction from 2015 to 2021 utilized PROMs, with a majority (73.7%) having used the BREAST-Q [110,111,112]. The further development of ERAS pathways relies on the comparison of preoperative approaches, surgical techniques, and postoperative management, contingent on the availability of subjective PROM data.

The conceptualization of breast reconstruction from a clinical standpoint should include not only surgical concerns and physical outcomes. Regardless of satisfaction with surgical outcomes, studies have shown that patients can suffer significant emotional and physical distress throughout the process, affecting patient self-image and self-esteem and carrying the potential of contributing to significant psychosocial morbidity, including anxiety and depression [110,111,112]. Carr et al. explored the support needs of patients following breast reconstruction, describing postsurgical psychosocial needs as multifaceted, requiring not only provider support but also broader social support to help patients rebuild their spiritual, social, and psychological wellbeing [113]. Their study, along with others, has shown the benefit of support groups in improving body image, reducing feelings of isolation and stigma, and improving community advocacy and shared decision-making [114,115] Beyond psychosocial improvements, support groups facilitated by trained medical professionals have been shown to improve access to information regarding recovery among patients who have undergone breast reconstruction in addition to improving quality of life and coping skills more broadly through connection, empowerment, and education [116].

Furthermore, with the previously described role of technology in medicine, it is of paramount importance to utilize technological means to improve patient education, accessibility to breast reconstruction, treatment adherence, and postoperative management. Telemedicine has emerged as a promising avenue through which to improve all these metrics. As previously described, many patients undergoing mastectomy do not pursue reconstruction either due to lack of accessibility, financial burden, knowledge, or misinformation [117]. Telemedicine has been described in the literature as a mechanism that can facilitate increased pre-mastectomy consultation and second opinion visits and has been shown to reduce the number of preoperative visits before surgery [118]. Moreover, the National Coalition for Cancer Survivorship Telehealth Project found that telehealth was an acceptable method for seeking second opinions, treatment monitoring, mental health visits, and postoperative monitoring, with benefits including reduced cost for patients, increased convenience, and reduced anxiety [119]. Further on the topic of cost, Stearns et al. found that while telehealth was previously not widely covered by insurance, it is now widely covered at the same or reduced rates compared with in-person visits [120]. Research has shown no significant differences in BREAST-Q scores between cohorts that received in-person versus telehealth care, both for preoperative consultations and postoperative follow-up visits, coupled with cost savings of over USD 1500 in the telehealth group [121]. A majority of patients have generally found telemedicine to be easy to use, reporting that they would use it again in the context of breast reconstruction [122].

## 5. Conclusions

Since their conception over two decades ago, ERAS pathways have transformed perioperative care by emphasizing faster recovery, reducing complications, and improving patient outcomes. Now, the focus of ERAS has shifted towards optimizing the patient experience by focusing on patient education and long-term recovery strategies aimed at restoring patients to their preoperative form, physically, emotionally, and functionally. Given the emerging data demonstrating the favorable outcomes associated with certain approaches to analgesia, surgical techniques, and postoperative management, it is crucial that further investigation of these evidence-based measures leads to broader adoption, eventually contributing to a refined standard of care. To maximize their impact, ERAS pathways must continue to expand beyond the immediate perioperative period by incorporating digital tools, validating patient-reported outcome measures like BREAST-Q, and providing structured psychosocial support to address the burden of breast reconstruction.

In reframing ERAS from a provider and hospital-centered algorithm to a patient-focused and evidence-based pathway, we propose adopting a new framework: Prepare, Protect, Restore, and Empower. With this reimagined pathway, we aim not only to improve surgical outcomes but also to reframe breast reconstruction as a process driven by the patient experience.

## Figures and Tables

**Table 1:** The core principles of ERAS in breast reconstruction, adapted by Temple-Oberle et al. [18].

Period/Purpose	Item	Recommendation
Preoperative	Preadmission Counseling	Provide detailed preadmission counseling regarding expectations
	Preadmission Optimization	≥1 month smoking and alcohol abstinence; BMI ≤ 30 kg/m^2^
	Perforator Flap Planning	CTA as required
	Fasting	Minimize fasting; permit clear fluids up to 2 h before surgery
	Carbohydrate Loading	Maltodextrin-based fluids administered 2 h before surgery
Prophylaxis	Venous Thromboembolism (VTE)	Assess risk; maintain low molecular weight/unfractionated heparin ± mechanical methods
	Infection	Chlorhexidine skin preparation; intravenous broad-spectrum antibiotics
	Nausea/Vomiting	Antiemetic medications
Intraoperative	Pain Management	Multimodal analgesia
	Anesthesia	Total intravenous anesthesia (TIVA)
	Preventing Hypothermia	Forced air; temperature monitoring for maintenance ≥ 36 °C
	Fluid Management	Use of balanced crystalloid solutions and pressors
Postoperative	Pain Management	Opioid-sparing, multimodal analgesia
	Early Feeding	Oral food and fluids within 24 h
	Flap Monitoring	Frequent monitoring during the first 72 h; implantable Doppler devices for buried flaps
	Wound Management	Conventional sutures for incisional closure; debridement and negative pressure for complex wounds
	Early Mobilization	Ambulation within 24 h
	Post Discharge Support	Early physiotherapy, supportive care

**Table 2:** Current evidence of outcomes associated with ERAS utilization in IBR, implant-based breast augmentation, and ABR.

Authors	Procedure	Outcomes Associated with ERAS
Niu et al. (2023) [20]	IBR	No significant differences in complications in same-day discharge cohort vs. overnight cohort
Taylor et al. (2024) [21]	IBR	↓ LOS, postoperative pain, and postoperative opioid use
Kennedy et al. (2020) [22]	IBR	↓ LOS and postoperative opioid use
Hatchell et al. (2024) [23]	IBR	↓ Unplanned readmission
Gehring et al. (2024) [24]	IBR	No significant differences in complications in same-day discharge cohort vs. overnight cohort
Stahl et al. (2024) [25]	Implant-based elective augmentation	↑ BREAST-Q scores
Seren et al. (2024) [26]	Implant-based elective augmentation	Early discharge with rapid return to daily activities
Wong et al. (2023) [27]	Implant-based elective augmentation	↓ Postoperative opioid use
Offodile et al. (2019) [28]	ABR	↓ LOS and postoperative opioid use
Astanehe et al. (2018) [29]	ABR	↓ LOS, postoperative opioid and emetic use, postoperative pain, and time to resume regular diet and activity
Linder et al. (2022) [16]	ABR	↓ LOS and surgical time
Muetterties et al. (2023) [30]	ABR	↓ LOS and opioid use
Haddock et al. (2024) [31]	ABR	↓ LOS and opioid use
Oh et al. (2018) [32]	ABR	↓ Cost
Bajaj et al. (2024) [33]	ABR	↓ Cost
Kaoutzanis et al. (2018) [34]	ABR	↓ LOS, opioid use, and cost

IBR = implant-based reconstruction; ABR = autologous reconstruction; LOS = length of stay; BREAST-Q = standardized questionnaire to assess patient-reported outcome measures; ↓ denotes a decrease; ↑ denotes an increase.

**Table 3:** A revised ERAS model for breast reconstruction, structured around patient experience-driven pillars.

Pillar	Focus	Examples
Prepare	Predict risk and optimize baseline health	Web applications, AI/ML * risk scoring
Protect	Minimize intraoperative morbidity	Robotic DIEP harvest, pectoral nerve block
Restore	Enhance functional and sensory recovery	Neurotization, ciNPT ^†^
Empower	Support psychosocial and long-term recovery	PROM, Telehealth, survivor groups

* AI/ML = artificial intelligence/machine learning. ^†^ ciNPT = closed incision negative pressure therapy.

## Data Availability

All data included in this narrative review are publicly available.
